# Significantly different noun-verb distinguishing mechanisms in written Chinese and Chinese sign language: An event-related potential study of bilingual native signers

**DOI:** 10.3389/fnins.2022.910263

**Published:** 2022-10-26

**Authors:** Lewen Xu, Tao Gong, Lan Shuai, Jun Feng

**Affiliations:** ^1^Department of Psychology, Hangzhou Normal University, Hangzhou, China; ^2^Zhejiang Key Laboratory for Research in Assessment of Cognitive Impairments, Hangzhou, China; ^3^Institutes of Psychological Sciences, Hangzhou Normal University, Hangzhou, China; ^4^School of Foreign Languages, Zhejiang University of Finance and Economics, Hangzhou, China; ^5^Google, New York, NY, United States; ^6^Educational Testing Service, Princeton, NJ, United States

**Keywords:** deaf signers, event-related potential, Chinese sign language, part-of-speech, noun-verb dissociation

## Abstract

Little is known about: (a) whether bilingual signers possess dissociated neural mechanisms for noun and verb processing in written language (just like native non-signers), or they utilize similar neural mechanisms for those processing (due to general lack of part-of-speech criterion in sign languages); and (b) whether learning a language from another modality (L2) influences corresponding neural mechanism of L1. In order to address these issues, we conducted an electroencephalogram (EEG) based reading comprehension study on bimodal bilinguals, namely Chinese native deaf signers, whose L1 is Chinese Sign Language and L2 is written Chinese. Analyses identified significantly dissociated neural mechanisms in the bilingual signers’ written noun and verb processing (which also became more explicit along with increase in their written Chinese understanding levels), but not in their understanding of verbal and nominal meanings in Chinese Sign Language. These findings reveal relevance between modality-based linguistic features and processing mechanisms, which suggests that: processing modality-based features of a language is unlikely affected by learning another language in a different modality; and cross-modal language transfer is subject to modal constraints rather than explicit linguistic features.

## Introduction

Aural-oral languages, referred generally to both written and spoken forms of languages (Capek et al., 2009), are dependent extremely on the distinction of noun and verb categories. The neural mechanisms of native speakers’ noun and verb processing, indicated mainly by functional Magnetic Resonance Imaging (fMRI) and electroencephalogram (EEG), have long been a focus in neurolinguistics (see Moseley and Pulvermüller, 2014; Feng et al., 2019 for reviews). Some studies argued that there lacked clear-cut dissociation between aural-oral languages’ noun and verb processing, based on findings of similar and widely distributed activations of brain areas in both verb and noun processing (e.g., Tyler et al., 2001; Li et al., 2004; Momenian et al., 2016). However, a large amount of studies have repetitively and consistently reported that understanding nouns and verbs in many aural-oral languages induced differentiated neural mechanisms: verbs are usually processed in the left inferior frontal and/or middle temporal area, whereas noun processing generally activates left temporal and/or parietal areas (Damasio and Tranel, 1993; Gerfo et al., 2008); verb processing tends to involve somatic motor cortex of human mirror neuron system, while noun processing does not (Gallese and Lakoff, 2005; Binder and Desai, 2011); and verbs often evoke significantly larger waveforms of event-related potentials (ERPs, e.g., P200, N400, and P600) than nouns (Preissl et al., 1995; Feng et al., 2019).

Meanwhile, as to what factor, syntax or semantics, leads to generally conformed noun-verb dissociation has been of great controversy (see Moseley and Pulvermüller, 2014; Xia et al., 2016 for reviews). Syntactic factors are usually attributed by studies using artificial words and/or morphologically altered words as stimuli (e.g., Tyler et al., 2004; Shapiro et al., 2005), while semantic factors are usually considered essential by studies performing semantic-relatedness judgment task (e.g., Liu et al., 2007; Xia and Peng, 2022) or semantic decision tasks at a sentence level (e.g., Lee and Federmeier, 2006; Moseley and Pulvermüller, 2014; Feng et al., 2019). Furthermore, existing findings are based predominately on normal hearing and/or brain-damaged subjects, whose native languages are unanimously aural-oral. This makes it inadequate to demonstrate whether the noun-verb dissociation is a general mechanism in human language processing, or just a modality-based feature.

Compared to aural-oral languages, sign languages (SLs) belong to a completely different modality, and more importantly, it is very hard to conclude a general criterion for classifying part-of-speech (POS) for SLs (Schwager and Zeshan, 2008). Although the nouns and verbs in some SLs [e.g., American Sign Language (ASL), German Sign Language (GSL)] could be distinguished comprehensively by their language-specific morphology, semantic and syntax (Schwager and Zeshan, 2008), or by means of different ratios of mouthing, sizes of signs, durations of signings, frequencies of movement, and measures of movement [as in Israeli Sign Language, Tkachman and Sandler (2013)]. This is because morphological markers are basically employed in the SLs whose corresponding aural-oral languages rely heavily on inflections (ASL-English, GSL-German, etc.), while most SLs put semantically close-related signs adjacently or do not use morphological markers at all [e.g., Chinese Sign Language (CSL)]. Syntactically, in most SLs, a sign can be used as either an argument or a predicate without any formal marking. Most significantly, in most SLs, one sign usually contains both the meaning of an action and that of one or a few entities of the same origin. To native signers, the meanings of the action(s) and their entities have no explicit differences, e.g., “book(s)” and “to read a book,” “airplane” and “to fly,” or “food” and “to eat” in CSL. However, whether native signers possess separate neural mechanisms between nouns and verbs during CSL processing remains unclear. It is also controversial whether CSL essentially has no POS, or native signers could implicitly distinguish nouns and verbs in CSL but could not explicitly represent the difference (as it is rather difficult to express abstract ideas in CSL).

Noting these, by investigating the neural mechanisms of noun and verb processing during native signers’ CSL understanding, we can clarify whether POS exists in CSL or not. Meanwhile, exploring signers’ noun-verb mechanisms in a written language (WL) as L2, i.e., written Chinese^1^ for Chinese signers, also helps examine whether learning a L2 from another modality tends to influence L1’s original neural representations, e.g., whether improvement in the level of written Chinese can affect native signers’ neural mechanisms for nominal and verbal meaning processing in CSL. Moreover, the findings can be used to study whether semantic differences can trigger the noun-verb neural distinction in aural-oral languages. Using Chinese sentences with the construct of NP (noun phrase) + *mei* (没, “no”) + target word (verb/noun), Feng et al. (2019) have claimed that semantic factors play an essential role in the neural dissociation between noun and verb processing in native Chinese speakers. As Chinese is famous for its paucity in morphological markers and *mei* can appear before a noun or a verb without hinting its part-of-speech (Lv, 1980; Zhu, 1985), participants can only determine, via semantics, whether a word was used as a verb or a noun in a sentence. Therefore, if distinct neural mechanisms between noun and verb processing are evident in signers’ comprehension of written Chinese as L2, it would advocate that the neural distinction is due to semantic differences inherent to nouns and verbs. In addition, if native signers do not show significantly distinct ERP patterns between nominal meaning and verbal meaning processing in sign sentences, it would also indicate that the noun-verb neural dissociation in aural-oral languages is more likely caused by semantic factors, because a sign often contains both the nominal and verbal meanings, whose semantics are seldomly considered different in native signers’ eyes. In other words, no semantic differences exist within a sign, so the corresponding noun-verb neural dissociations would no longer exist.

Previous studies have touched upon the interaction between the two language modalities and corresponding neural mechanisms, usually from macro aspects (MacSweeney et al., 2008), e.g., comparing neural substrates of facial expressions during speech production between deaf native signers and hearing non-signers (Pyers and Emmorey, 2008), and between hard-of-hearings and hearing non-signers in their uses of co-speech gestures (Casey and Emmorey, 2008) and verbal descriptions of spatial relationships (Emmorey et al., 2005). However, more essential linguistic differences caused by modal constraints (e.g., POS) have hardly been addressed when exploring cross-modal language transfer and its neural mechanisms. In addition, early studies typically compared neural mechanisms, respectively, for native signers and native non-signers to understand their native languages. However, the question of whether learning a L2 from another modality would affect L1’s processing mechanisms can be investigated more precisely by recruiting participants who master both languages and examining their neural mechanisms for the processing of related but significantly different, modality-based linguistic features of the two languages. According to Kelly and Barac-Cikoja (2007) and Fu (2020), the reading abilities of deaf students are generally four years behind those of hearing students. Meanwhile, the learning of written Chinese in native signers is much harder than learning CSL in Chinese non-signers. Possible reasons for this are that CSL contains rather few abstract meanings as a visual language, and its signing rate (about 70 signs per minute) is much less than the speech rate in Mandarin Chinese (about 245 syllables per minute, note that most Mandarin Chinese words are disyllabic), which lead to less consumption of cognitive resources for non-signers to understand CSL. Therefore, the influence from written Chinese as L2 to CSL as L1 is more explicit and easier to observe. In addition, the process of L2 improvement in native signers (written Chinese as L2) typically lasts longer than that in non-signers (CSL as L2), which makes it conducive to observing the impact of increase in L2 on L1’s neural mechanisms. All these suggest that native deaf signers with written language abilities are no doubt ideal participants for investigating the abovementioned issues.

Noting these, we designed the current study to investigate the following three research questions:

(a) Whether bilingual signers have dissociated neural mechanisms, respectively, for noun and verb processing in written language and for nominal and verbal meanings in sign languages;
(b) Whether such neural mechanisms are consistent with the two language’s remarkable differences on reliance of POS concept; and
(c) Whether learning of L2 from another modality influences L1’s processing mechanism.

We recruited Chinese deaf signers with different levels of written Chinese understanding abilities, designed two comprehension tasks, respectively, in written Chinese and CSL, and obtained and analyzed the ERP signals considered useful to bring forth rather direct and immediate results of POS understanding. As consistently reported in previous studies (Preissl et al., 1995; Liu et al., 2007; Xia et al., 2016 *inter alia*), neural dissociation between verbs and nouns in aural-oral languages has been through the major stages of sentence processing and can be observed by significantly larger amplitudes evoked by verbs than those by nouns (indicating that verb understanding requires more cognitive resources than noun understanding). The major stages of sentence processing include: (a) an initial identifying period of word categories, which can be reflected by the ERP component of P200, occurring ∼100-300 ms after target onset (Preissl et al., 1995; Liu et al., 2007); (b) a computation period of verb (predicate) and nouns (arguments), which can be reflected by N400, occurring ∼300-500 ms after target onset (Federmeier et al., 2000; Lee and Federmeier, 2006); and (c) a final top-down semantic integration period of the whole sentence’s components, which can be reflected by P600, occurring ∼500-800 ms after target onset (Liu et al., 2008; Xia et al., 2016). The region-of-interests (ROIs) and effects used in sign language ERP studies have been basically the same as those used in aural-oral language ERP studies, i.e., the frontal and frontotemporal areas for P200 and P600, the temporal to parietal areas for N400 (Kutas et al., 1987; Neville et al., 1997; Capek et al., 2009). It is worth noting that when signers process corresponding written language as L2, they show similar P200, N400 and P600 effects to those of non-signers in their processing of written language as L1 (Skotara et al., 2012; Mehravari et al., 2017). Meanwhile, the effects shown in signers’ L2 processing were also considered to be evoked by the predication of target word’s lexical form/orthography (P200), the semantic violation of target word’s meaning in the sentence (N400), and the syntactic incongruity caused by target word (P600) (Neville et al., 1997; Mehravari et al., 2017). Accordingly, we chose the analogous ROIs design widely adopted in previous studies for both reading tasks of written Chinese and CSL experiments in the current study. To be specific, the frontocentral sites were set as the ROI of P200 and P600, and the centroparietal sites as the ROI of N400.

With a short duration, P200 is usually claimed to represent a highly automated initial identification period of language processing (Holcomb et al., 1992; Barber et al., 2004), such as the initial recognition of part-of-speech information (Friederici et al., 1999). Previous studies have revealed that during the P200 phrase, the amplitude revoked by verb processing is generally larger (more positive) than that of noun processing, as the semantics within verbs are more complicated than the semantics within nouns, the latter of which can be automatically detected by native speakers during the initial lexical recognition stage (Preissl et al., 1995; Federmeier et al., 2000; Liu et al., 2007). Some research also considered P200 as a marker of the extent of expectancy for certain items (Viswanathan and Jansen, 2010), whereas many noun-verb processing studies hardly confirmed P200’s suggestibility to the POS of a particular target word, regardless of whether priming words were included in the experimental materials (Preissl et al., 1995; Liu et al., 2007; Xia et al., 2016).

The N400 effect has long been remarked as an index of lexical/semantic processing, such as the computation of semantic relationship between predicate and its arguments (Federmeier et al., 2000; Lee and Federmeier, 2006). Previous studies concerning noun-verb dissociation usually pointed out that verb processing tended to evoke significantly larger (more negative) N400 amplitude than that of noun-processing, as verb semantics is more complicated (thus consuming more cognitive resources) than noun semantics, i.e., during the N400 period, nouns as verbs’ thematic roles (who does what to whom) are assigned to verbs, and a verb’s semantics has to be handled first by calculating the number of its arguments (Friederici, 2011).

Finally, P600 represents two rather than one unitary phenomenon (Friederici et al., 2002): syntactic violation during sentence comprehension is represented by the generally reported centroparietal-originated P600, while the frontocentral-originated P600 typically reflects semantic integration. In this study, the centroparietal P600 is not expected as syntactically violated sentences were not used as stimuli. Although P600 component is normally expected and reported in the centroparietal area (e.g., Coulson et al., 1998; Federmeier et al., 2000) to indicate the extent of syntax violation, distinct P600 in the frontocentral area between Chinese noun and verb processing was reported (Xia et al., 2016) to reflect integrating complexity (Feng et al., 2019). Furthermore, the target words’ part-of-speech can only be clarified by integrating the target words’ meaning with other components in the sentences. Therefore, the frontocentral area was selected as the ROI of P600 in this research.

In our study, we conducted two experiments to examine our research questions among signers with different written Chinese levels. In experiment 1, the recruited signers were asked to comprehend 200 written Chinese sentences in the configuration of NP (noun phrase) + *mei* (“no”) + target words (noun/verb). In experiment 2, the recruited signers were asked to comprehend 200 CSL sentences with the same meanings and orders as those in experiment 1. Our working hypotheses lie in two aspects. First, we assume that the target verbs and nouns would cause significantly distinct ERP effects of P200, N400 and P600 in experiment 1, and the separated patterns would become more explicit with the increase of native signers’ written Chinese levels. Since the materials and sentence structure in experiment 1 ensure that the signers can only judge the target words’ part-of-speech by their semantics, the essential role of semantic factors in distinguishing nouns and verbs can be identified if there exists noun-verb neural dissociation. Second, we anticipate null dissociated neural mechanisms in processing nominal and verbal meanings in CSL, i.e., no noun-verb dissociated patterns exist in CSL’s P200, N400 and P600 effects, regardless of the native signers’ written Chinese proficiency. There are no POS criteria observed in CSL, such as no subtle articulating differences in ratios of mouthing, sizes of signs, durations of signs, or frequencies of movements, and neither morphological markers nor rigid syntax are found to exist in CSL. Therefore, for signers, there is no necessity to distinguish the formal differences of target signs or the roles of target signs used as in sentences. If the above assumptions are validated by experimental results, we can safely conclude that learning L2 from a different modality is unlikely to affect L1’s original processing mechanism.

## Materials and methods

The experimental protocol of this study was approved by the Institutional Review Board (IRB) of the Center for Cognition and Brain Disorders, Hangzhou Normal University. The methods were carried out in accordance with the approved guidelines. Informed consents were obtained from all participants.

### Participants

We recruited 60 deaf bilingual signers (28 males, 32 females, whose ages ranged from 17 to 20 years old, mean = 18, SD = 1.5) from a local deaf high school (Hangzhou, China) for the study. These participants had CSL as their native language and written Chinese as their second language. All the deaf participants were from families with hearing parents and started learning sign language in deaf pre-school at age 3 to 4 (mean = 3.5, SD = 0.29). The participants’ Age of Acquisition (AoA) of written Chinese ranged from 4 to 5 (self-report), mean = 4.4, SD = 0.5. According to their average scores of a written Chinese comprehension test in the recent semester (the total score is 32), they were divided into three level groups: 20 participants whose average scores over 24 were assigned to the high L2 level group; 20 with average scores between 17 and 24 were assigned to the mid (intermediate) L2 level group; and the remaining 20 with average scores between 9 and 16 were assigned to the low L2 level group. The comprehension test is a major component of the Chinese final exam for senior students within local deaf schools. Four kinds of questions with significantly different levels of difficulty (easier, easy, neutral and hard, each having 8 points) were used in the test.

Each of the easier questions (four questions, two points each) asked the students to choose between two options (A and B) a more suitable one for a given sentence, e.g.:

英雄是_____ 的人。(*Heroes are who*_____.)

A. 视死如归 (*face death unflinchingly*) B. 班门弄斧 (*teach a fish how to swim*)

All the participants answered all the easier questions correctly.

Each of the easy questions (four questions, two points each) asked whether two words with identical shape had the same meaning, e.g.:

A. 这是一朵很漂亮的花。(*This a flower which is very beautiful*.)
B. 钱不可以乱花。(*Do not spend money too casual.*)

All the participants with mid and high L2Level correctly judged the meanings of this part, while the signers with low L2Level had a mean score of 6.1 (SD = 1.2).

Each of the neutral questions (four questions, two points each) asked the students to choose the correct conjunctive word for each blank in many sentences, such as:

这次地震没有造成伤亡, ____损失还是很严重的。 ____今年经济压力很大, 我们还是 ____把地震损失补回来, ____保证经济平稳发展。

(*The earthquake caused no casualties, ____ the loss is still serious. ____the economy is under a lot of pressure this year, we have to ____make up for the earthquake damage, ____ensure the economy stably develop.*)

A. 然而(*whereas*)   尽管(*although*)   既要(*not only*)   也要(*but also*)
B. 同时(*simultaneously*)   虽然(*though*)   但是(*but*)   甚至(*even*)
C. 不仅(*not only*)   而且(*but also*)   特别(*particularly*)   因此(*therefore*)
D. 虽然(*though*)   但是(*but*)   尤其(*especially*)   从而(*hence*)

All the low L2Level signers scored 0 in this part; those with mid L2Level had a mean score of 4.6 (SD = 0.91); and those with high L2Level had a mean score of 7.7 (SD = 0.73).

The sole hard question (8 points) asked the students to correctly arrange a number of words in a sentence (only the complete, accurate alignment was awarded the full point; otherwise, no point):

这是_ _ _ _ _ 电影院。  (*This is _ _ _ _ _ cinema*.)

A. 现代化的(*modern*)    B. 杭州市 (*Hangzhou’s*)    C. 唯一的 (*only*)

D. 新型的 (*new typeof*)    E. 一座 (*one*)

All the signers with low and mid L2Level scored 0 in this question, while the signers with high L2Level had a mean score of 6 (SD = 3.5).

Based on these facts, we consider that the reliability and validity of the written Chinese comprehension test are reasonable, so is the L2Level division used to group signers. Moreover, there was no significant effect of AoA on the signers’ Chinese comprehension scores [one-way ANOVA: *F*(1,59) = 0.893, *p* = 0.538, η^2^ = 0.138].

All the participants had a hearing loss > 90 dB, they were strongly right-handed as tested by the handedness inventory (Snyder and Harris, 1993), and they had normal or corrected-to-normal visions and no history of neurological diseases. They voluntarily participated in the study and were paid a proper remuneration after completing it.

### Materials

The stimuli used in this study were first adopted in Feng et al. (2019)’s experiment, which demonstrated that the neural mechanisms were distinct between noun and verb processing in native non-signers. In the current study, we used the same set of stimuli to further investigate whether deaf bilingual signers also had separated neural mechanisms between written noun and verb processing, just like hearing non-signers. This research used semantically correct sentences (both in the written and sign modalities) as stimuli. Due to the absence of morphological markers in written Chinese and CSL, bilingual signers could only distinguish target part-of-speech (nominal meaning or verbal meaning) after reading/viewing the whole sentence and figuring out its meaning in the sentence. Therefore, target word/sign was put at the end of each sentence, and possible hints on target part-of-speech were also excluded.

All the signers participated in the two experiments successively: Exp. 1 was a comprehension task of written Chinese, the materials consisted of a total of 200 written Chinese sentences. Exp. 2 was a comprehension task of CSL, the materials included 200 CSL sentences having identical meanings and “word” orders to those in Exp. 1.

In Exp. 1, the 200 sentences had an identical construction of NP (noun phrase) + *mei* (‘no’) before the final target words. Table 1 shows some examples of the sentences. The complete list of the sentences is shown in Supplementary Table 1 in Supplementary materials.

**TABLE 1:** Examples of Chinese sentences used as materials in Exp. 1.

	NP	mei	target words	
1	这台相机(This camera)	没(no)	胶卷(films)	Noun
2	这个猎人(This hunter)	没(no)	狗(dogs)	Noun
3	这辆单车(This bike)	没(no)	座椅(saddle)	Noun
4	这个西瓜(This watermelon)	没(no)	糖分(sugar)	Noun
5	这名球员(This player)	没(no)	技术(skills)	Noun
6	这个季节(This season)	没(no)	大风(gale)	Noun
7	这条金鱼(This goldfish)	没(no)	牙齿(teeth)	Noun
8	这部电影(This film)	没(no)	声音(sound)	Noun
9	这颗卫星(This satellite)	没(no)	天线(antenna)	Noun
10	这家饭店(This restaurant)	没(no)	啤酒(beers)	Noun
11	这群候鸔(These migratory birds)	没(not)	回来(return)	Verb
12	这只兔子(This rabbit)	没(not)	跳(jump)	Verb
13	这列火车(This train)	没(not)	到达(arrive)	Verb
14	这场大雨(This heavy rain)	没(not)	停止(stop)	Verb
15	这颗子弹(This bullet)	没(not)	爆炸(explode)	Verb
16	这家银行(This bank)	没(not)	破产(rupt)	Verb
17	这架飞机(This plane)	没(not)	起飞(take off)	Verb
18	这个男生(This boy)	没(not)	抽烟(smoke)	Verb
19	这个婴儿(This infant)	没(not)	哭闹(cry)	Verb
20	这只乌鸦(This crow)	没(not)	喝水(drink)	Verb

Ten of them have verbs as their final target words and the rest ten have nouns as their final target words.

Among the 200 sentences, 100 used verbs as the final target words, and the rest used nouns. Each target word was used exactly once. According to the Modern Chinese Dictionary (Lv and Ding, 2012), the target verbs and nouns in these sentences can hardly be used as other POS, and they have unique interpretations in most native speakers. All the target words are rather high frequency words according to the corpus by the Ministry of Education of the People’s Republic of China,^2^ which contains more than 150,000 Chinese characters (the exact number of words is incalculable, as Chinese characters are often combined rather freely to make words). According to the corpus, the mean log-transformed (base *e*) frequency of the selected verbs is 0.113‰ (SD = 0.177‰), and that of the selected nouns is 0.111‰ (SD = 0.152‰). One-way ANOVA reported no significant differences between the selected verbs and nouns [*F* (7,92) = 0.103, *p* = 0.732, η^2^ = 0.003]. Besides word frequency, the Chinese character frequency is another factor that needs to be considered (Yan et al., 2006). Character frequency was based on the times that a character appeared in 1,000 words, and if a character’s frequency is greater than 1, it is a high frequency character according to Wang et al. (1986). No significant difference was found between frequencies of the target nouns’ initial (Mean = 1.95, SD = 0.62) and last characters (Mean = 1.71, SD = 0.59), and the target verbs’ initial (Mean = 1.85, SD = 0.56) and last characters (Mean = 1.48, SD = 0.61) in the one-way ANOVA test [*F* (3,391) = 0.478, *p* = 0.7, η^2^ = 0.004]. We also examined whether the stroke numbers of the target nouns and verbs would cause any potential influence. The one-way ANOVA results showed that: the stroke numbers of the target nouns (Mean = 16.15, SD = 4.955) and target verbs (Mean = 17.25, SD = 4.482) had no significant difference [*F* (1, 198) = 2.710, *p* = 0.101, η^2^ = 0.014].

Meanwhile, to avoid potential influence by different target words’ predictabilities, ten hearing non-signers from Hangzhou Normal University were recruited to perform a cloze test. In this test, the ten non-signers were told to read each NP + *mei* construct used in Exp. 1, and to write down any possible final word (a noun or a verb) which first came to their minds for completing the sentence. Data of this predictability test showed that the mean predictability of all the target nouns in Exp. 1 was 7.6% (SD = 13.4%), and which for all the target verbs was 10.1% (SD = 18.6%). One-way ANOVA reported no significant differences between predictabilities of the selected verbs and nouns [*F* (7,92) = 1.512, *p* = 0.173, η^2^ = 0.103], indicating the target words’ predictabilities were all rather low and would not influence the experiments significantly.

According to Chinese grammar, *mei* and *bu* are the two mostly used negation words (Lv, 1980; Zhu, 1985). However, *bu* is almost exclusively (though not entirely) used to negate verbs. While, *mei* is rather free to be appeared before verbs and nouns. Considering the dominance of *mei* and *bu* as negation words in Chinese, and *bu* merely matching nouns, it is rather important for *mei* to negate verbs and nouns freely, and we seldom found reports of *mei*’s preference. Also, we compared the numbers of verbs and nouns which were written down by the ten non-signers who performed the above cloze test (for analyzing the target words’ predictabilities). Among the total 2,000 words written down (ten non-signers, each closed 200 sentences of Exp.1), there were 1,037 verbs, 946 nouns and 17 noun-verb ambiguous words, consistent with the grammatical feature of *mei*, i.e., using before verbs and nouns without hinting the POS. Therefore, the *NP* + *mei* + *target word* construct ensures that bilingual signers can only understand these sentences by analyzing sentential semantics. Such a sentence structure is also common and legitimate in CSL. In Exp. 2, the 200 sign sentences had the same “word” order as the written sentences in Exp. 1. The word order in CSL is more fixed than that in Chinese sentences (Lv, 2017), though is not completely fixed. NP + target noun/verb + *mei* is also logical in CSL, as well as in Chinese, which is usually used to emphasize the sentence-final word *mei*, to indicate that a certain state does not exist or something has not happened. Each of the CSL stimuli was presented in a videoclip, which was performed at a natural signing speed (about 70 signs per minute) by a CSL interpreter with normal hearing.

Twenty native Chinese speakers were recruited to rate the difficulty of the meanings of the 200 written sentences by a five-point scale: very simple, simple, neutral, hard, and very hard to understand. All the target sentences were rated as very simple to understand.

Another 20 deaf signers with different levels of written Chinese understanding (7 of them with low L2Level, 7 with mid L2Level, and 6 with high L2Level) and not participating in the formal experiments were recruited to rate the meaning difficulty of the 200 written sentences and the 200 sign sentences. It is worth noting that there are two main differences between sign and written sentences, namely word order and ellipsis. Therefore, the 20 deaf signers were also asked to rate the rationality of the CSL sentences, by a five-point scale (very rational, rational, neutral, irrational, very irrational).

As for difficulty, the sign sentences were all rated as “very simple,” and one-way ANOVA test showed no significant effect of L2Level (*p* = 0.214) on rating the written sentence difficulties. As for rationality, about 80% of the written sentences were rated as “very simple,” and the rest as “simple.” All the target CSL sentences were rated as “very rational”. This could be due to the facts that: all the target sign sentences were basically very simple and short, thus making it difficult to express their meanings by ellipsis; and the construct of NP + *mei* + target word is also common in CSL. All these suggest that the meaning difficulty of the target words and signs, as well as the rationality (including sign order and ellipses) of the CSL sentences would not greatly affect the experimental results, and the deaf signers’ written Chinese understanding levels would not influence their understanding of CSL sentences.

In addition to rating the sentences, we asked one half of the 20 deaf signers to read all the 200 written sentences in Exp. 1, and then, all the 200 sign sentences in Exp. 2, and the other half to read all the 200 sign sentences prior to the 200 written sentences. We found that when the signers watched the video clip first, they tended to automatically use signs to help understand written sentences in the next task; while reading written sentences first would not induce such influence. Noting these, we fix the task order in the formal experiments as silently reading all the written sentences (Exp. 1) before reading (viewing) all the sign sentences (Exp. 2).

### Procedure

Each bilingual signer participated in both experiments. The experiments were carried out in a soundproof and electrically shielded room, where participants sit comfortably in front of a 17-inch CRT screen. The screen resolution was set to 1024 × 768, the distance between the screen and participants’ eyes was approximately 100 cm. All characters in the written sentences were displayed on the screen in the font of Song, with a size of 60 pixels for being seen clearly; the resolution of the CSL videoclips was 720p, and these videoclips were presented in a full screen mode. Participants were told to try remaining quiet and still throughout the experiments. All the participants were informed to use only the language presented in the current sentence, not the other one while thinking or translating.

In many ERP studies on signers’ reading performance, a slower presentation rate was used in reading tasks (Foucart and Frenck-Mestre, 2011, 2012; Tanner et al., 2013, 2014). For example, in the procedure of Mehravari et al’s. (2017) study, a blank screen appeared first for 1000 ms, which was followed by a 500 ms fixation cross and a 400 ms inter-stimulus interval. Then, each single stimulus word appeared for 600 ms, with a 200 ms interval between words. After the final word of the sentence was shown for 600 ms, there was a 1000-ms blank screen for signers to make grammatical/semantic judgment. The presentation rate in this procedure was slower than the presentation rate typically used in many ERP studies of L1 users (Mehravari et al., 2017). In our study, we used an even slower presentation rate and there was no judgment task for the deaf participants.

**FIGURE 1:**
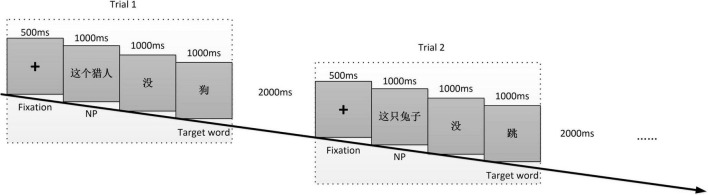
The procedure of Exp. 1.

In Exp. 1, there are totally 200 semantically and logically correct Chinese sentences, 100 of which ended with verbs, and the other 100 ended with nouns. All the 200 sentences had a consistent word order as: NP (noun phrase) + *mei* + target verb or noun. The procedure of each trial was shown in Figure 1. In each trial, a crossing fixation first appeared for 500 ms at the center of the screen to catch participants’ attention. Then, the stimulus sentence was shown in the center of the screen in the sequence of NP, 没 (*mei*), and target word, e.g., 这个猎人 (This hunter), 没 (no), and 狗 (dog), duration of each was 1000 ms. A 2000 ms interval was shown after the final target word and before the next crossing fixation. Each sentence was shown in one trial.

**FIGURE 2:**
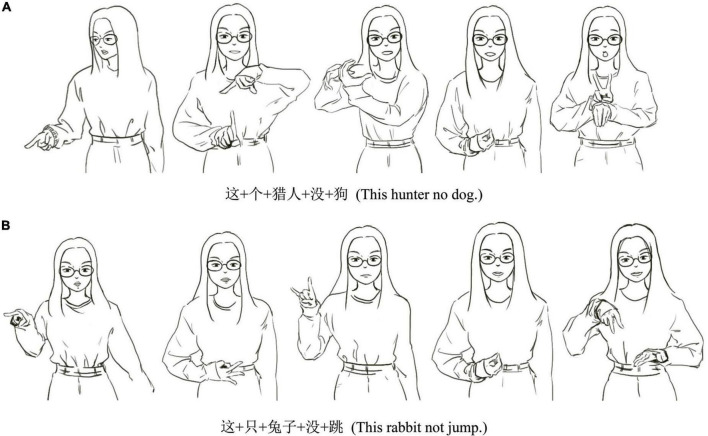
Illustrated examples of the CSL stimuli, which were performed by an interpreter.

The procedure of Exp. 2 is consistent with the one of Exp. 1, except that each sign sentence was presented in a single videoclip at a normal signing rate (approximately 70 signs per minute). Each sentence was shown in one trial. Figure 2 shows two examples of the CSL stimuli performed by the interpreter. The total 200 sentences in each experiment were divided into 20 blocks, each block contained 10 sentences. In each block, there were 5 trials ended with nouns (or corresponding signs translated from written sentences ended with nouns) and 5 trials ended with verbs (or corresponding signs translated from written sentences ended with verbs). Trials appeared randomly in a block. Participants were allowed to take a 20-s inter-block break. Each experiment lasted about 20 min.

Due to the following facts, the two experiments employed silent reading and viewing tasks but not semantic decision task which has been used more in sentence processing research. Firstly, the reading ability of deaf students is weaker than that of hearing students (Kelly and Barac-Cikoja, 2007), generally four years behind (Fu, 2020), and the participants in this research also contained a group of deaf students with rather low reading abilities. If semantic decision task was used, 200 semantically incorrect sentences need to be added as fillers, then the deaf signers’ overall duration for experiments would increase significantly, which would be more likely to make experimental results affected by the degree of fatigue of the subjects. Secondly, in terms of attention and visual field, the words displayed in the center of the screen are central information, while the signs successively presented in the screen are extrafoveal information which are more relied upon by deaf signers (Dye et al., 2008; Bélanger et al., 2012). If a decision task is added to the written language comprehension that is more difficult to rely on extrafoveal information, it may further cause the deaf subjects to consume more cognitive resources in Experiment 1 than in Experiment 2, thus leading to potential problems. Besides, the use of silent reading tasks is not rare in ERP studies (Brown et al., 2000; Peiyun et al., 2018). In summary, we used silent reading and viewing tasks in the two experiments.

### Electroencephalogram recording and preprocessing

We used a Neuroscan Synamps2 system for EEG data recording, and the software Scan 4.5 provided by NeuroScan, Inc. for data analysis. During each experiment, the participants wore a Quick-Cap 64 elastic cap for data recording. We located one pair of electrodes above and below each participant’s left eye for the VEOG signal and another pair outside outer canthi of both eyes for the HEOG. The signals from all recordings were referenced to the signals of the left mastoid online and re-referenced to the averaged signals of the left and right mastoids offline. Each electrode’s impedance was kept below 5k Ω. The band pass filtering was set between.05 and 100 Hz with the sample frequency of 1,000 Hz. Ocular movements and other artifacts were excluded from analysis by setting the threshold at ±100 μV. Baseline correction was performed during the time window of –100 to 0 ms before the stimuli onset. All the ERP data were computed over a range of 200 ms before and 800 ms after the onset of stimulus (i.e., the final target word).

**FIGURE 3:**
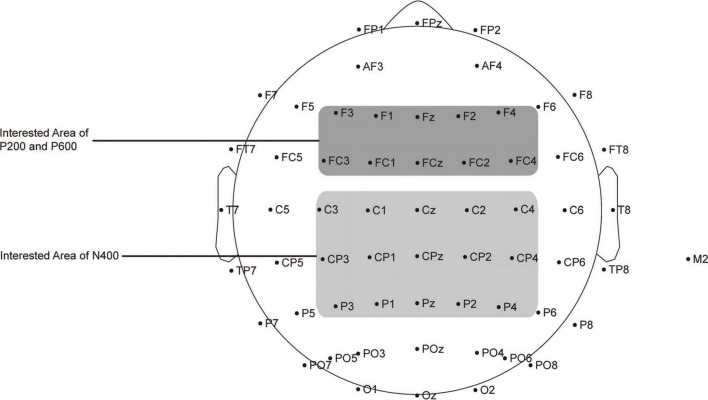
Distribution of the 64 electrodes over the scalp used to record EEG signals. The electrodes used to capture P200 and P600 are marked in dark gray, and those used to capture N400 are marked in light gray.

The ERP data for analysis were recorded online through different ROIs. According to previous studies, we focused on three ERP components relevant for both sign and aural-oral language processing: P200 (ranging from 100 to 300 ms), N400 (300 to 500 ms) and P600 (500 to 800 ms). As shown in Figure 3, the signals from the electrodes Fz, FCz, F3, F4, F1, F2, FC3, FC4, FC1 and FC2 were used to calculate the average volatilities of P200 (Friederici et al., 2002; Zhang et al., 2003; Liu et al., 2007, 2008), and those from Cz, CPz, Pz, C3, C4, C1, C2, CP3, CP4, CP1, CP2, P3, P4, P1 and P2 were used to calculate the average volatilities of N400 (Kutas and Federmeier, 2000; Liu et al., 2007). And those from Fz, FCz, F3, F4, F1, F2, FC3, FC4, FC1 and FC2 were selected as the ROI as (frontocentral) P600 effect. As mentioned earlier, P600 has two main forms, a common P600 within centroparietal area reflecting syntax violation (e.g., Coulson et al., 1998; Federmeier et al., 2000), and a frontocentral P600 representing semantic integration (Friederici et al., 2002; Xia et al., 2016). In this study, all the stimuli sentences were syntactically plausible. Meanwhile, the final target words’ part-of-speech were masked by the negation word *mei* (没), so the participants could only identify the part-of-speech by integrating the target words’ meaning with the rest parts of the sentence. Therefore, the frontocentral P600 for semantic integration was expected instead of the centroparietal P600 for syntax-repair.

### Data analysis

We adopted a 2 × 2 × 3 (modality × POS × L2Level) design in the two separate experiments to examine: (a) whether native signers showed dissociated ERP effects between understanding Chinese nouns and verbs; and (b) whether the ERP effects were influenced by native signers’ Chinese proficiencies. There were three types of independent factors: two within-subject variables, namely modality (with two levels: SL and WL) and POS (with two levels: verb and noun, verb is the base),^3^ and one between-subject variable, namely L2Level [with three levels: low, mid (intermediate), and high, for simplicity, this ordinal categorical variable is treated as an ordinal variable].

For each of the three ERP components (P200, N400 and P600) obtained in the SL and WL experiments, we fixed two mixed-effects regression models. In the first model, the average amplitude of the ERP component over the time window obtained in the WL experiment was treated as the dependent variable, and POS, L2Level, and their two-way interaction were treated as the independent variables. In the second model, the average amplitude of the ERP component obtained in the SL experiment was treated as the dependent variable, and POS, L2Level, and their two-way interaction were treated as the independent variables. We controlled the family wise Type I error probability by setting the critical *p* value for identifying significant effects as.05/9 ≈0.005, here, 9 was set according to three types of data (P200, N400, P600) multiplies three types of independent variables (POS, L2Level, and their two-way interaction). We conducted simple effects test whenever a significant two-way interaction was reported in order to interpret the influence of such interaction on the ERP components. Such test could inform whether the noun’s (or verb’s) P200/N400/P600 waveform becomes larger along with the increase of signers’ L2Level.

In each mixed-effects model, we incorporate a random intercept, i.e., the subject ID, to partial out influence on results from individual difference. Compared to simple linear models or ANOVA tests, the mixed-effects models allow for simultaneous consideration of multiple covariates, while keeping the between-individual (family and region) variance under statistical control (Baayen, 2008). Although maximal random effect structures involving random slopes are theoretically desirable (Barr et al., 2013) and have been applied in recent individual difference studies (e.g., Protopapas et al., 2007), such complicated models were not pursued here in consideration of practical constraints on model convergence (Bates et al., 2015). In our study, the mixed-effects models were implemented using the lme4 (Bates et al., 2015) and lmerTest (Kuznetsova et al., 2017) packages in R.

## Results

### Event-related potential data on written Chinese

The statistic results of P200, N400, and P600 during the written sentence processing are summarized below (detailed statistics are in Supplementary Table 2 in Supporting materials).

**FIGURE 4:**
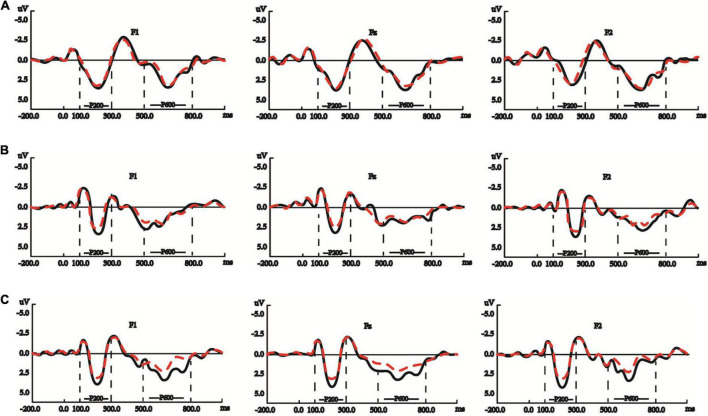
**(A)** Native signers with low L2Level did not possess significantly dissociated P200 or P600 effect between processing noun (red dashed lines) and verb (black solid lines) in written Chinese sentences. The y-axes in these panels are negative up. **(B)** Native signers with mid L2Level possessed significantly dissociated P200 but not P600 effect between processing noun (red dashed lines) and verb (black solid lines) in written Chinese sentences. The y-axes in these panels are negative up. **(C)** Native signers with high L2Level possessed significantly dissociated P200 and P600 effects between processing noun (red dashed lines) and verb (black solid lines) in written Chinese sentences. The y-axes in these panels are negative up.

During the P200 (100-300 ms) time window of written sentence processing, the model showed that: neither POS (verb/noun) (β = −0.519, Std. Error = 0.363, *t* = −1.430, *p* = 0.158) nor L2Level (β = 0.093, Std. Error = 0.131, *t* = 0.711, *p* = 0.479) had significant main effects, but the interaction between the two was significant (β = 0.518, Std. Error = 0.168, *t* = 3.083, *p* = 0.003), and the P200 waveforms for verbs and those for nouns were getting more dissociated along with the increase in native signers’ L2Level. The graphs in Figure 4 show the three groups of signers’ averaged P200 waveforms, respectively, activated by the target nouns and verbs, 100–300 ms after target onset.

The simple effects test further showed that: L2Level within verb was significant (*p* < 0.001), but L2Level within noun was not (*p* = 0.741), and P200 waveforms evoked by verbs became larger along with the increase in native signers’ L2Level; POS within low L2Level was not significant (*p* = 0.566), as shown in Figure 4A, and POS within mid L2Level (*p* = 0.01) and high L2Level (*p* = 0.004) were significant, as shown in Figures 4B,C.

**FIGURE 5:**
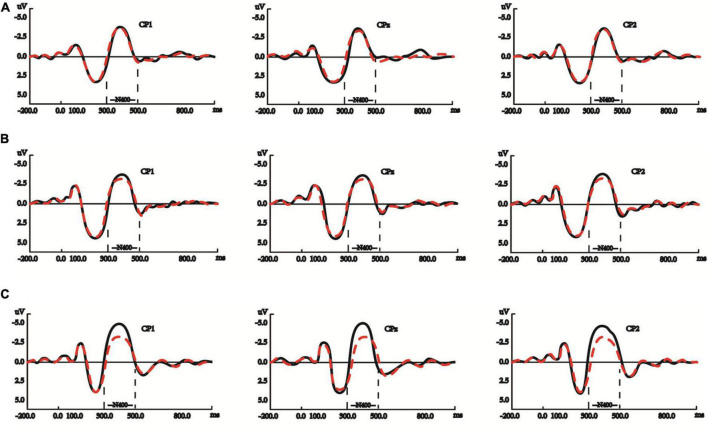
**(A)** Native signers with low L2Level did not possess significantly dissociated N400 effect between processing noun (red dashed lines) and verb (black solid lines) in written Chinese sentences. The y-axes in these panels are negative up. **(B)** Native signers with mid L2Level did not possess significantly dissociated N400 effect between processing noun (red dashed lines) and verb (black solid lines) in written Chinese sentences. The y-axes in these panels are negative up. **(C)** Native signers with mid L2Level possessed significantly dissociated N400 effect between processing noun (red dashed lines) and verb (black solid lines) in written Chinese sentences. The y-axes in these panels are negative up.

During the N400 (300–500 ms) time window of written sentence processing, the ANOVA showed that: POS (β = 0.534, Std. Error = 0.182, *t* = 2.939, *p* = 0.005) had a significant main effect, but not L2Level (β = 0.085, Std. Error = 0.072, *t* = 1.176, *p* = 0.242), and the interaction between the two was significant (β = −0.419, Std. Error = 0.084, *t* = −4.987, *p* < 0.001), and the N400 waveforms for verbs and those for nouns were getting more dissociated along with the increase in native signers’ L2Level. The graphs in Figure 5 show the three groups of signers’ averaged N400 waveforms, respectively, activated by the target nouns and verbs, 300–500 ms after target onset.

The simple effects test further showed that: L2Level within verb was significant (*p* < 0.001), but L2Level within noun was not (*p* = 0.125), and the N400 waveforms evoked by verbs became larger along the increase in native signers’ L2Level; POS within low (*p* = 0.273) or mid L2Level (*p* = 0.027) were not significant, as shown in Figures 5A,B, but POS within high L2Level was significant (*p* < 0.001), as shown in Figure 5C, which conforms to the fact that native signers with high L2Level showed significantly distinguished N400 waveforms between nouns and verbs.

During the P600 (500–800 ms) time window of written sentence processing, the ANOVA showed that: POS had a significant main effect (β = −0.476, Std. Error = 0.211, *t* =-2.254, *p* = 0.028), but not L2Level (β = 0.004, Std. Error = 0.072, *t* = 0.048, *p* = 0.961), and the interaction between POS and L2Level had a significant effect (β = 0.407, Std. Error = 0.098, *t* = 4.162, *p* < 0.001), and the P600 waveforms for verbs and those for nouns were getting more dissociated along with the increase in native signers’ L2Level. The graphs in Figure 4 show the three groups of signers’ averaged P600 waveforms, respectively, activated by the target nouns and verbs, 500-800 ms after target onset.

The simple effects test further showed that: L2Level within verb was significant (*p* < 0.001), but L2Level within noun was not (*p* = 0.686), and there was sustained P600 increase in amplitude for verbs along with the increase in native signers’ L2Level; POS within low or mid L2Level were not significant (*p* = 0.468, *p* = 0.022), as shown in Figures 4A,B, and that within high L2Level was significant (*p* < 0.001), as shown in Figure 4C, which conforms to the significantly dissociated P600 waveforms between nouns and verbs in the participants from the high L2Level group.

### Event-related potential data on Chinese sign language

The statistic results of P200, N400 and P600 during the CSL sentence processing are listed below (the detailed statistic results are in Supplementary Table 3 in Supporting materials).

During the P200 time window of the CSL processing, no main effect was found for POS (β = 0.050, Std. Error = 0.082, *t* = 0.609, *p* = 0.545) or L2Level (β = 0.023, Std. Error = 0.028, *t* = 0.818, *p* = 0.415), and the interaction between the two was also not significant (β = 0.014, Std. Error = 0.038, *t* = 0.377, *p* = 0.707).

During the N400 time window of the CSL processing, no main effect was found for POS (β = −0.040, Std. Error = 0.217, *t* = −0.182, *p* = 0.856) or L2Level (β = 0.101, Std. Error = 0.071, *t* = 1.424, *p* = 0.157), and the interaction between the two was also not significant (β = −0.044, Std. Error = 0.100, *t* = −0.434, *p* = 0.665).

During the P600 time window of the CSL processing, no main effect was found for POS (β = 0.130, Std. Error = 0.222, *t* = 0.585, *p* = 0.559) or L2Level (β = −0.097, Std. Error = 0.073, *t* = −1.336, *p* = 0.184), and the interaction between the two was also not significant (β = 0.032, Std. Error = 0.103, *t* = 0.309, *p* = 0.758).

**FIGURE 6:**
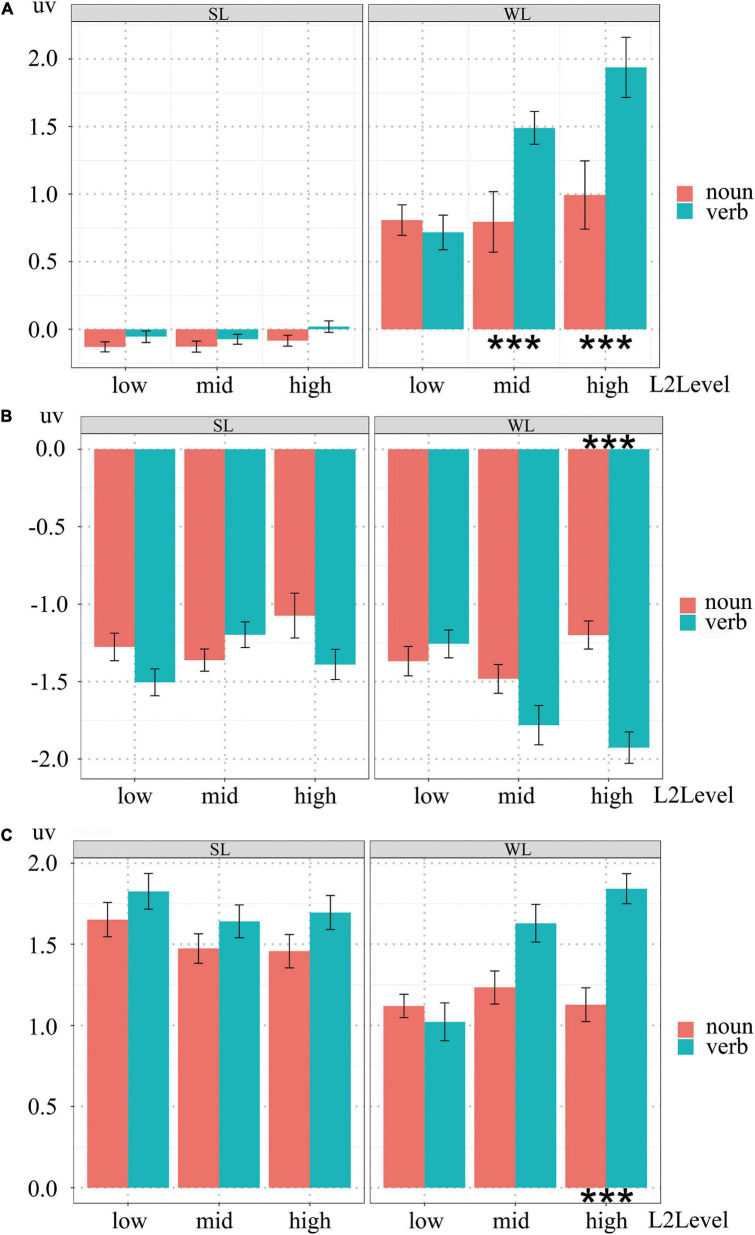
**(A)** The dissociation of P200 effect between nouns and verbs in the three groups of native signers with different L2Level. Black stars indicate that signers with mid and high L2Level possessed significantly different P200 effect between processing nouns and verbs in written Chinese sentences (*p* < 0.006). **(B)** The dissociation of N400 effect between nouns and verbs in the three groups of native signers with different L2Level. Black stars indicate that signers with high L2Level possessed significantly different N400 effect between processing nouns and verbs in written Chinese sentences (*p* < 0.006). **(C)** The dissociation of P600 effect between nouns and verbs in the three groups of native signers with different L2Level. Black stars indicate that signers with high L2Level possessed significantly different P600 effect between processing nouns and verbs in written Chinese sentences (*p* < 0.006).

Figures 6 A–C showed the different extents of noun-verb ERP dissociations in the three groups of native signers’ CSL and written Chinese sentence understanding, respectively.

## Discussion

### Noun-verb neural dissociation in written language and the group differences

Though a few studies have reported that there hardly exist a clear-cut between noun and verb processing (Li et al., 2004; Momenian et al., 2016), more studies have concluded that the neural mechanisms between noun and verb processing are significantly different during aural-oral language understanding (e.g., Damasio and Tranel, 1993; Gerfo et al., 2008; Xia et al., 2016). In addition, previous studies focused mainly on the aural-oral language modality and native speakers with normal hearing, but rarely touched upon a completely different modality of human language (i.e., sign languages) and native signers with severe hearing loss. Therefore, it is far from enough to draw a universal conclusion about dissociated neural mechanisms between nominal and verbal meaning in human language, especially with respect to most sign languages that rather hard to distinguish part-of-speech. The current study is the first one that attempts to explore the neural mechanisms between noun and verb processing by recruiting deaf bilingual signers (CSL as L1 and written Chinese as L2) with different levels (high, middle, low) of written Chinese understanding (Exp. 1). Our results showed that these bilingual signers possessed significantly dissociated ERP effects between nouns and verbs (as shown by the significant main effect of POS on N400 and P600 in the WL experiment), just like hearing native speakers, and such neural separation became continuously more explicit with the increase in the bilingual signers’ levels of written Chinese understanding (as shown by the significant two-way interactions between POS and L2Level in the three models from the WL experiment and the results of the simple effects tests). These results are consistent with Chinese language’s rather high dependence on using part-of-speech. Furthermore, it remains controversial which factor, semantics or syntax, is more essential to the observed noun-verb neural distinction (e.g., Shapiro et al., 2005; Moseley and Pulvermüller, 2014; Xia et al., 2016). Studies using lexical decision tasks with artificial words and/or morphologically altered words basically concluded that syntax factors are crucial. If the combination of two characters that can form a target Chinese word is incongruous, it would also cause syntactic violation of the sentence (Wang et al., 2008). By contrast, the importance of semantic factors have been highlighted in studies adopting sentential semantic decision tasks (Lee and Federmeier, 2006; Moseley and Pulvermüller, 2014), especially in Chinese language, which seldom contains morphological markers and inflections (Xia et al., 2016; Feng et al., 2019). In Exp. 1, syntactic properties were excluded, and the semantic construct (NP + *mei* + noun/verb) ensured that the native signers could only identify the final target words’ part-of-speech based on their semantics in the sentences (*mei* did not provide any specific hints to the target words’ part-of-speech). Therefore, we consider that the noun-verb distinguishing results in Exp. 1 is mainly due to semantic factors.

The above dissociated noun-verb ERP patterns in P200, N400 and P600 were not identical among the three groups of deaf participants. As shown in Figure 6, dissociated noun-verb P200 effects were only evident in signers with mid and high L2Level, while dissociated noun-verb N400 and P600 effects were found only in signers with high L2Level. Such group differences were caused partially by different requirements for cognitive resources in the three main stages of sentence comprehension, and possibly also caused by different exposure to written Chinese.

In Exp. 1, during the 100-300 ms time window (the P200 period), with the increase in the level of written Chinese understanding, verb processing elicited increasingly more positive P200 waveforms in bilingual signers, whereas noun processing did not. That suggests, bilingual signers gradually possessed dissociated neural mechanisms between nouns and verbs in understanding written Chinese. Here, the low L2level bilingual signers did not exhibit dissociated neural mechanisms between noun and verb processing, but in both the mid and high-level groups, verb processing evoked significantly larger P200 waveforms than noun processing. P200 is generally considered to reflect automatic recognition of lexical categories in the early stages of language understanding (Federmeier et al., 2000; Liu et al., 2007). In our view, the characteristics of P200 indicate its low demand for cognitive resources, since at this early stage of sentence processing, rather complicated processing such as calculating the relationships between words or integrating background knowledge within/beyond context has not been performed. This low demand of cognitive resources is also consistent with the generally believed “high degree of automation” of P200 (Preissl et al., 1995; Feng et al., 2019). Therefore, as long as the deaf signers’ Chinese proficiency is not particularly insufficient, the initial lexical recognition can still be completed automatically with less cognitive resources, conforming to the null main effect of POS nor L2Level but significant interaction between the two. This is supported by the rather low scores in the native signers with low L2Level (not reaching the half of the full score, as shown in Section “Participants”), while comparative studies have pointed out that most hearing students about 4 years younger than deaf students can complete the corresponding reading test correctly (e.g., Fu, 2020). Our results are also in line with such difference between hearing individuals and native signers during sentence processing. Moreover, these findings explicitly revealed that as their Chinese proficiency increased, bilingual signers’ automatic distinction between nouns and verbs improved synchronously. Unlike nouns, the rather complicated semantics involved in verbs (e.g., Gentner, 1982; Snedeker and Gleitman, 2004) became detected gradually more automatic and fluent (Preissl et al., 1995; Feng et al., 2019), which led ultimately to the trigger of explicit noun-verb distinction, i.e., verb processing evoked larger P200 waveforms than noun processing, and the gap between verb’s P200 waveform and noun’s P200 waveform was expanding. As in aural-oral languages, nouns are often used for rather quiescent state like objects or concepts; by contrast, verbs are usually used to express dynamic state, like actions and what subjects do to objects. In our experiment, the increased level of written Chinese understanding made bilingual signers more aware of the different semantics between nouns and verbs during the initial sentence processing period, and hence, they started to show gradually separated P200 waveforms between verb and noun processing in written Chinese.

During the 300–500 ms time window (the N400 period), bilingual signers with low or middle L2Level did not show explicitly separated neural mechanisms between noun and verb processing in written Chinese, but those with high L2Level showed a significant neural dissociation between noun and verb understanding, as verbs elicited significantly larger N400 waveforms than nouns. Meanwhile, as bilingual signers’ L2Level increased, verbs rather than nouns started to elicit more negative waveforms. Comparing to the P200 period, significantly more complex processing has taken place in the N400 period, such as revealing certain words’ semantics, numbers, arguments, and expecting possible match (Lee and Federmeier, 2006; Friederici, 2011). In other words, the N400 phase corresponds to a bottom-up process, in which a sentence’s integral meaning is constituted by each word. In the process of sentence comprehension with relatively simple semantic and syntactic relations, the N400 stage can even become the final stage of sentence processing (Friederici, 2011). During this period, verb’s semantics is usually handled first by counting the number and identities of its arguments, and then, nouns as verbs’ thematic roles (who does what to whom) are assigned to verbs (Lee and Federmeier, 2006; Friederici, 2011). It has been uniformly agreed that more cognitive resources have to be allocated for verb understanding due to rather complex semantics of verbs, which results in larger N400 amplitude (Xia et al., 2016; Feng et al., 2019). As cognitive resources can only be called more effectively by higher proficient L2Level, only the group of native signers with the highest Chinese proficiencies showed significantly dissociated N400 effects between nouns and verbs.

Note that many studies (e.g., Holcomb et al., 1999; West and Holcomb, 2000; Hagoort, 2003) have revealed a wrap-up effect or sentence-ending global effect, which evokes rather complex ERP waveforms (also known as N400-like or N700) than N400 and P600. However, such wrap-up effect was reported primarily in sentence processing tasks involving some syntactic violations, as such violations would keep participants (re)considering the syntactic structure of the whole sentence even after the appearance of the final target word. By contrast, such wrap-up effect was seldom reported in other research using sentences involving semantic issues, such as context relatedness or word congruency (Federmeier and Kutas, 1999; Hagoort and Brown, 2000; Borovsky et al., 2010). Since the sentences used in our study were semantically correct and did not contain syntactical violations, using sentence-final words as targets would not cause potential wrap-up problems.

During the 500-800 ms time window (the P600 period), the high L2Level group of signers showed significantly larger P600 waveforms for verb processing than for noun processing, more explicit than those from the other two groups did not. This indicates that, with the increase of written Chinese understanding, bilingual signers gradually showed dissociated neural mechanisms between nouns and verbs. In-depth integration of sentence components is a key process which taken place during the P600 period of sentence processing, and contextual clue and background knowledge have to be taken into account for comprehensive understanding, the top-down analysis also has to be performed to eliminate semantic ambiguities (Hoeks et al., 2004; Kuperberg et al., 2007). As these operations are further supplemental to those in the N400 stage and calling for rather large number of cognitive resources, it is not surprising that only the deaf participants with the highest L2Level possessed the corresponding dissociated P600 effects between nouns and verbs. It is worth noting that some previous studies have argued that there also exists a syntax-related P600, often evident around the centroparietal area and reflecting whether any syntactic violation occurred (e.g., Osterhout et al., 2002). The P600 effects in our study reflected only in-depth semantic integration but not syntactic repair, because in our study, the written sentences having NP + *mei* + noun or verb as final target words did not contain any syntactic violation.

Meanwhile, as the bilingual signers’ written Chinese proficiencies increase, it is very likely that their exposure to written Chinese also becomes longer, thus making them more familiar with the target Chinese characters. Since P200 is considered associated with the pattern recognition of physical stimuli (Federmeier et al., 2000; Liu et al., 2007), N400 to indicate the expectation of certain matches (Friederici, 2011; Kutas and Federmeier, 2011), and P600 to reflect the prediction of some common sense related to the subjects, the bilingual signers’ increase in the familiarities of target characters inevitably make them more sensitive in the above processings.

Overall, the above ERP results consistently indicate that, within the major stages of language processing, the improved written Chinese proficiency makes bilingual signers, to a greater extent, capable of distinguishing the inherent differences between noun and verb semantics. For example, nouns often refer to lifeless objects or living entities, they are relatively more stable and less affected by time change, they can serve as the subject or object of a verb, as well as the object of a preposition, and they are typically used to express a theme. By contrast, verbs often describe actions or states, they are relatively more dynamic and sensitive to time change, and usually employed to discuss a theme. Along with the improvement in L2Level, the bilingual signers are getting progressively more skilled at identifying the inherent difference between noun and verb semantics, from the early stage of lexical processing to the later bottom-up semantic integration, till the final top-down reanalysis. Many linguistic and cognitive studies have also discussed the inherent difficulties of verbs (e.g., Gentner, 1982; Snedeker and Gleitman, 2004); e.g., the sense of a verb represents not only an action itself, but also its subject (and object), duration, method and path, outcome, etc., and native speakers can hardly understand verbs without these semantic elements, which are also far more complicated than those embedded in nouns. Many developmental studies have also showed that ever since children’s language acquisition, verbs are harder to learn than nouns (e.g., Gentner, 1982; Gentner and Boroditsky, 2001). Even in languages like Korean, in which verbs often appear at the end of a sentence, children’s acquisition of verbs tends to be later than that of nouns (Choi and Bowerman, 1991). As shown in Gillette et al. (1999), after a video which contained a related action to the artificial verb or noun, adults were sometimes more confused about how to exactly demarcate a verb’s sense than a noun’s, as shown by the fact that their accuracy of guessing an artificial noun’s meaning was much higher than that of guessing an artificial verb’s. As far as we can see, the reason why identifying verbs is of more importance also lies in the fact that a verb is usually the core of an aural-oral sentence. Without identifying the verb’s subject/object, method, path, duration, outcome, etc., a sentence cannot be understood correctly. Furthermore, research concerning embodied-cognition also claimed that if one cannot imagine the verb’s action meaning, as well as the above-mentioned semantic elements, one cannot understand the whole sentence completely; and processing verbs needs to activate not only Broca’s area but also motor cortex (human mirror neuron system) for the action’s virtual stimulation, while such mechanism was not evident in noun processing (Gallese and Lakoff, 2005; Boulenger et al., 2006).

### Undissociated nominal-verbal mechanism in sign language

In Exp. 2, we testified that the native signers did not have significantly different ERP effects between their processing of nominal and verbal meanings in CSL, no matter how their Chinese proficiency increased.

Generally, it is rather difficult to conclude reliable methods of dividing POS in SLs, though nouns and verbs in a few SLs (like ASL and GSL) can be comprehensively distinguished by morphological markers, semantic nuances and syntactic structures (Schwager and Zeshan, 2008), or by subtle characteristics like different ratios of mouthing between nouns and verbs, different manners of sign movement (continuous for verbs, restrained for nouns), and different durations and sizes of signing (verbs longer and/or larger than nouns) (Tkachman and Sandler, 2013). However, these factors seldom exist in CSL: no morphological markers, strict correspondence for a sign and its syntactic position, nor duration/size differences are used. Most importantly, whether the meaning of a sign represents actions or entities makes no difference in CSL native signers, because a CSL sign usually contains both verbal and nominal meanings of the same origin. Therefore, we claim that the null noun-verb dissociation of CSL in Exp. 2 conforms one of the essential and modality-based features of CSL, i.e., POS barely exists in CSL.

The P200 effect was seldom reported in SL processing, significantly dissociated P200 waveforms between nominal and verbal meanings were not found in Exp. 2, either. These null effects are consistent with the main characteristics of sign languages, i.e., not containing part-of-speech. During this initial stage of language processing, the bilingual signers generally do not automatically distinguish between nominal and verbal meanings, which is not affected by their long-term Chinese learning. This is correlated with the fact that a sign in CSL (as well as in most other SLs) usually contains both homologous verbal and nominal meanings which usually have the same origin, and native signers do not treat them differently at all (Schwager and Zeshan, 2008). Hence, it is neither possible nor necessary for the signers to differentiate the verbal and nominal meanings in signs during the P200 period.

No significantly different N400 effects were found between nominal and verbal meaning processing, regardless of participants’ levels of written Chinese understanding. This still conforms to the inherent feature of lacking part-of-speech in CSL. In our opinion, unlike the significant integrating difficulties in verbs than in nouns (Gentner, 1982; Snedeker and Gleitman, 2004), there are two main factors that make integrating sign semantics rather simple: first, most meanings of signs can be directly perceived through viewing; and second, the sentential orders of sign languages basically ensure that signs with close-related semantics be placed adjacently. Therefore, compared to aural-oral languages, fewer cognitive resources are needed for sentential integration in sign languages, thus leading to the observed undissociated N400 effects in Exp. 2.

During the P600 period, the final stage of sentence processing, no significant difference was evident between nominal and verbal meaning in native signers’ CSL understanding, regardless of L2Levels. CSL sentences usually put the topics and/or the most important semantics at the beginning. Meanwhile, long sentences are seldom used. These constructive characteristics of CSL sentences make it unnecessary to allocate additional cognitive resources for the top-down reanalysis during the P600 period, thus resulting in the identical P600 waveforms during native signers’ CSL processing.

In summary, through the typical ERP components reflecting the major stages of language understanding, regardless of bilingual signers’ written Chinese understanding levels, there were no significantly dissociated neural mechanisms between processing of CLS’s nominal meaning and verbal meaning, which was in line with the most striking feature of CSL, i.e., lacking explicit criterions for part-of-speech.

In our opinion, these results also indicate that semantic factors (the inherent semantic differences between nouns and verbs) play an essential role in dissociated noun-verb neural mechanisms. Given that each target sign contains both nominal meaning(s) and verbal meaning(s) with the same origin, native signers do not need to distinguish the semantic differences within each sign to identify its part-of-speech, which leads to the null neural dissociation between nominal and verbal meaning understanding.

### The null cross-modal influence from L2 to L1’s processing mechanism

In general, the above results collectively show that the dissociated neural processing between nouns and verbs in aural-oral Chinese is a modality-based feature. Meanwhile, the issue of whether learning a second language in another modality would influence the learner’s neural mechanisms for his/her native language has been discussed in some previous studies. Most of them generally compared the neural substrates of facial expressions, co-speech gesture, or verbal descriptions of spatial relationship during the production of native languages of deaf signers and hearing non-signers (Emmorey et al., 2005; Casey and Emmorey, 2008; Pyers and Emmorey, 2008). By contrast, our study explores specifically the relationship between significantly modality-based linguistic differences and corresponding potential changes of neural mechanisms. Our results indicate that there seems to exist relevance between a language’s modality-based feature and its neural mechanism, which is not likely to be affected by learning a language from another modality and/or having totally different characteristics. For example, the deaf signers gradually possessed neural dissociation between verbs and nouns in written Chinese understanding along with the increase of written Chinese levels, but the neural mechanisms between nominal and verbal meanings in CSL understanding remained intact. Also, such mechanisms seem to be rooted deeply in native signers’ sign language understanding; no matter how close their neural mechanisms of written Chinese understanding to native hearing speakers are, the neural dissociation between nouns and verbs are not transferred to native signers’ CSL understanding.

Our results also suggest that the characteristics of language modality play decisive roles during language understanding and transfer. Compared to aural-oral languages, one of the most evident characteristics in sign languages is that it is very difficult to express abstract concepts (Namir and Schlesinger, 1978; Borghi et al., 2014). This is also why most sign languages do not rely on part-of-speech (a rather abstract concept), but use a way that, in the eyes of aural-oral speakers, would cause terrible ambiguities in communications (Namir and Schlesinger, 1978; Schwager and Zeshan, 2008). Such phenomenon is obviously modality-based. In addition, the results of Exp. 1 and Exp. 2 collectively reveal rather direct relationship between a language’s modality-based feature (different degrees of dependence on part-of-speech) and its neural mechanism (different degrees of neural dissociation between processing nouns/nominal meanings and verbs/verbal meanings). Furthermore, such relationship remains unaffected while learning a second language from another modality. As shown in our study, although native signers gradually possessed dissociated neural mechanisms for noun and verb understanding in written Chinese, their neural mechanisms for CSL’s nominal and verbal meaning processing remained basically undissociated.

Being more complicated than language transfer within the same modality, language transfer across modalities is seriously affected by modal constraints rather than explicit linguistic features such as pronunciation, vocabulary, or syntax. One of the inherent modal constraints of sign languages is its rather low signing rate, compared to the speech rate of aural-oral languages. The time of making a sign is much longer than that of articulating a word; for example, as in spoken Chinese, the average number of syllables per minute is about 245 (most Chinese words are disyllabic), while the natural signing rate in CSL is approximately 70 signs per minute, resulting in a smaller working memory span when using SLs (not 7 ± 2 but 5 ± 1 items, according to Boutla et al., 2004). Obviously, the syntax and semantics of a sign language have to be thematically prominent and constructed straightforwardly; otherwise, effective communication with a smaller working memory span would be rather unaffordable. This is closely related to some of the main features of sign languages, e.g., delivering most important meanings at the front, putting closely related signs adjacently, and preferring short sentences. This also means that signs must be simple, concrete, and contain as little implicit semantics as possible. Therefore, a sign containing both verbal and nominal meanings, rather than using part-of-speech as in the modality of aural-oral languages, is in fact an inevitable choice for effective communication without additional cognitive resources. In other words, whether using part-of-speech is determined primarily by the respective characteristics of a language’s inherent modal constraints.

Last but not least, although the silent reading/viewing task adopted in this study is not rare in ERP studies (e.g., Brown et al., 2000; Peiyun et al., 2018), tasks requiring more attentions from participants (e.g., semantic violation decision task) are expected in future studies to further explore the cross-modal ERP patterns between nouns/nominal meanings and verbs/verbal meanings.

## Conclusion

By contrasting processing of verbal and nominal meanings in Chinese Sign Language, as well as verbs and nouns in written Chinese, our study demonstrated that deaf bilingual signers showed similar neural dissociation between noun and verb processing in understanding written Chinese, just like native non-signers, and this neural dissociation between noun and verb processing increased with bilingual signers’ written Chinese understanding levels. However, the bilingual signers did not show separated neural mechanisms to process CSL’ nominal and verbal meanings, in line with one of the most significant properties in CSL, lacking part-of-speech. These results indicate that the dissociated neural processing between nouns and verbs in aural-oral Chinese is modality-based. As for aural-oral languages that rely heavily on part-of-speech, especially in distinguishing verbs from nouns, it is reasonable that users also possess correspondingly dissociated neural mechanisms between noun and verb processing; however, for sign languages that lack part-of-speech and signs usually have homologous verbal and nominal meanings, it is not surprising to see that signers do not possess significantly dissociated neural mechanisms between nominal and verbal meaning understanding. Moreover, there seems to be a rather direct relation between a language modality’s remarkable linguistic feature and specific neural mechanism, and such mechanism would hardly be affected by learning a second language from another modality, the corresponding linguistic feature of which is totally distinct. Accordingly, research of cross-modal language transfer needs to pay more attention to modality-based linguistic feature(s).

## Data availability statement

All datasets generated for this study are included in the manuscript and/or the Supplementary material.

## Ethics statement

The studies involving human participants were reviewed and approved by the Institutional Review Board (IRB) of the Center for Cognition and Brain Disorders, Hangzhou Normal University. Written informed consent to participate in this study was provided by the participants or their legal guardian/next of kin.

## Author contributions

JF designed the research. JF and LX performed the experiments. LX analyzed the data. TG and LS drew the figures. JF, LX, TG, and LS wrote the manuscript. All authors contributed to the article and approved the submitted version.

## Footnotes

1 The form of written Chinese is the same across all variants of Chinese (such as Mandarin and Cantonese). In addition, although there are different sign *dialects* across China, all educated native signers can learn and use CSL. In general, it is common to take sign language as deaf people’s L1 while written language as their L2, such as in Yang (2008), Skotara et al. (2012), and Schönström (2013) etc. Spoken Chinese and written Chinese are of the same language, there have very close correspondences between their linguistic structures like vocabulary and grammar. However, there are barely such correspondences between CSL and written Chinese. In addition, considering their significantly different modalities, they are two completely different languages. Furthermore, concerning the acquisition order, deaf signers usually begin to learn CSL during pre-school, and start learning written language much later.

2
www.cncorpus.org

3 Though CSL has no POS, we use this abbreviation here for the ease of description, as well as “verb” for verbal meaning and “noun” for nominal meaning in CSL, respectively.
