# Supplementary material for: Significantly different noun-verb distinguishing mechanisms in written Chinese and Chinese sign language: An event-related potential study of bilingual native signers
Source: Front Neurosci. 2022 Oct 26;16:910263. doi: 10.3389/fnins.2022.910263 (PMC9643713; doi:10.3389/fnins.2022.910263)

**Supplementary Materials**

**Table S1.** 200 Chinese sentences were used as materials in Exp. 1, 100 with verbs as final

target words and 100 with nouns as final targets words, divided into 20 blocks.

| 1 | 这台相机  This camera | 没  no | 胶卷  films | Noun |
| --- | --- | --- | --- | --- |
| 2 | 这个茶杯This teacup | 没  no | 把手  handle | Noun |
| 3 | 这辆单车This bike | 没  no | 座椅  saddle | Noun |
| 4 | 这个孩子This child | 没  no | 零钱  pocket money | Noun |
| 5 | 这些学生These students | 没  no | 书包schoolbags | Noun |
| 6 | 这本书  This book | 没  no | 图画pictures | Noun |
| 7 | 这张抽屉This drawer | 没  no | 钥匙  keys | Noun |
| 8 | 这件衣服This garment | 没  no | 扣子buttons | Noun |
| 9 | 这个经理This manager | 没  no | 助手assistants | Noun |
| 10 | 这个学生  This student | 没  no | 校服  uniforms | Noun |
| 11 | 这位画家  This painter | 没  no | 铅笔  pencils | Noun |
| 12 | 这个社区  This community | 没  no | 学校  schools | Noun |
| 13 | 这位老人  This old man | 没  no | 医保  healthcare | Noun |
| 14 | 这支钢笔  This pen | 没  no | 墨水  ink | Noun |
| 15 | 这个电源  This power supply | 没  no | 插座  sockets | Noun |
| 16 | 这个球队  This team | 没  no | 队长  captain | Noun |
| 17 | 这个农民  This farmer | 没  no | 种子  seeds | Noun |
| 18 | 这种产品  This product | 没  no | 市场  market | Noun |
| 19 | 这个楼层  This floor | 没  no | 空调  air conditioners | Noun |
| 20 | 这间屋子  This room | 没  no | 窗帘  curtains | Noun |
| 21 | 这盒电池  These batteries | 没  no | 电量  power | Noun |
| 22 | 这位猎人  This hunter | 没  no | 狗  dogs | Noun |
| 23 | 这个部落  This tribe | 没  no | 灯  lights | Noun |
| 24 | 这次画展  This exhibition | 没  no | 票  tickets | Noun |
| 25 | 这棵小树  This tree | 没  no | 树叶  leaves | Noun |
| 26 | 这条裤子  These pants | 没  no | 腰带  belt | Noun |
| 27 | 这个西瓜  This watermelon | 没  no | 糖分  sugar | Noun |
| 28 | 这座城市  This city | 没  no | 工地  construction sites | Noun |
| 29 | 这位作家  This writer | 没  no | 稿费  royalties | Noun |
| 30 | 这套家具  These furniture | 没  no | 餐桌  tables | Noun |
| 31 | 这个部门  This department | 没  no | 主任  director | Noun |
| 32 | 这根电线  This electrical wire | 没  no | 胶带  adhesive tape | Noun |
| 33 | 这名球员  This player | 没  no | 技术  skills | Noun |
| 34 | 这名将军  This general | 没  no | 士兵  soldiers | Noun |
| 35 | 这位员工  This employee | 没  no | 能力  ability | Noun |
| 36 | 这双皮鞋  These leather shoes | 没  no | 鞋带  laces | Noun |
| 37 | 这个村庄  This village | 没  no | 村长  chief | Noun |
| 38 | 这片土地  This land | 没  no | 庄稼  crops | Noun |
| 39 | 这对夫妻  This couple | 没  no | 孩子  children | Noun |
| 40 | 这位护士  This nurse | 没  no | 手套  gloves | Noun |
| 41 | 这家餐厅  This restaurant | 没  no | 音乐  music | Noun |
| 42 | 这首古诗  This ancient poem | 没  no | 标题  headline | Noun |
| 43 | 这种语言  This language | 没  no | 文字  words | Noun |
| 44 | 这种材料  This material | 没  no | 香味  fragrance | Noun |
| 45 | 这个学校  This school | 没  no | 乐队  bank | Noun |
| 46 | 这头大象  This elephant | 没  no | 象牙  tusks | Noun |
| 47 | 这场比赛  This match | 没  no | 赢家  winners | Noun |
| 48 | 这名记者  This reporter | 没  no | 稿件  manuscript | Noun |
| 49 | 这副耳环  This pair of earrings | 没  no | 钻石  diamonds | Noun |
| 50 | 这只蜜蜂  This bee | 没  no | 翅膀  wings | Noun |
| 51 | 这个季节  This season | 没  no | 大风  gale | Noun |
| 52 | 这家餐馆  This restaurant | 没  no | 牛肉  beef | Noun |
| 53 | 这片森林  These forests | 没  no | 阳光  sunlight | Noun |
| 54 | 这名病人  This patient | 没  no | 病历  medical records | Noun |
| 55 | 这块手表  This watch | 没  no | 表带  strap | Noun |
| 56 | 这幢大厦  This building | 没  no | 窗户  windows | Noun |
| 57 | 这条金鱼  This goldfish | 没  no | 牙齿  teeth | Noun |
| 58 | 这只野猪  This wild boar | 没  no | 鬃毛  mane | Noun |
| 59 | 这部电影  This film | 没  no | 声音  sound | Noun |
| 60 | 这只箱子  This box | 没  no | 泡沫  foam | Noun |
| 61 | 这本词典  This dictionary | 没  no | 封面  cover | Noun |
| 62 | 这辆汽车  This car | 没  no | 汽油  petrol | Noun |
| 63 | 这个星球  This planet | 没  no | 生命  lives | Noun |
| 64 | 这家超市  This supermarket | 没  no | 橡皮  rubbers | Noun |
| 65 | 这个女生  This girl | 没  no | 对象  partner | Noun |
| 66 | 这剂中药  This TCM doze | 没  no | 疗效  effect | Noun |
| 67 | 这个瘦子  This lean person | 没  no | 脂肪  fat | Noun |
| 68 | 这个圆环  This ring | 没  no | 缺口  notch | Noun |
| 69 | 这种说法  This statement | 没  no | 依据  proof | Noun |
| 70 | 这种时候  This time | 没  no | 心情  mood | Noun |
| 71 | 这份套餐  This package | 没  no | 筷子  chopsticks | Noun |
| 72 | 这个国家  This country | 没  no | 铁矿  iron mines | Noun |
| 73 | 这种现象  This phenomenon | 没  no | 规律  law | Noun |
| 74 | 这家饭店  This restaurant | 没  no | 咖啡  coffee | Noun |
| 75 | 这条裤子  These pants | 没  no | 拉链  zippers | Noun |
| 76 | 这片草地  This grassland | 没  no | 蘑菇  mushrooms | Noun |
| 77 | 这个故事  This story | 没  no | 结局  ending | Noun |
| 78 | 这片沙漠  This desert | 没  no | 水源  water source | Noun |
| 79 | 这段山路  This mountain road | 没  no | 台阶  steps | Noun |
| 80 | 这片池塘  This pond | 没  no | 青蛙  frogs | Noun |
| 81 | 这颗卫星  This satellite | 没  no | 天线  antenna | Noun |
| 82 | 这个房间  This room | 没  no | 电视  television | Noun |
| 83 | 这门课程  This course | 没  no | 作业  homework | Noun |
| 84 | 这个学校  This school | 没  no | 宿舍  dormitories | Noun |
| 85 | 这家饭店  This restaurant | 没  no | 啤酒  beers | Noun |
| 86 | 这辆摩托  This motorbike | 没  no | 车轮  wheels | Noun |
| 87 | 这盒礼物  This present | 没  no | 茶叶  tea | Noun |
| 88 | 这个鱼钩  This fishhook | 没  no | 倒刺  barb | Noun |
| 89 | 这台电脑  This computer | 没  no | 密码  password | Noun |
| 90 | 这份试卷  This test paper | 没  no | 名字  name | Noun |
| 91 | 这个表格  This table | 没  no | 线条  lines | Noun |
| 92 | 这个篮球  This basketball | 没  no | 弹性  elasticity | Noun |
| 93 | 这块球拍  This racket | 没  no | 海绵  sponge | Noun |
| 94 | 这场比赛  This match | 没  no | 奖金  prize money | Noun |
| 95 | 这只天鹅  This swan | 没  no | 羽毛  feathers | Noun |
| 96 | 这名士兵  This soldier | 没  no | 武器  weapons | Noun |
| 97 | 这户人家  This family | 没  no | 邻居  neighbors | Noun |
| 98 | 这间教室  This classroom | 没  no | 钢琴  piano | Noun |
| 99 | 这个女生  This girl | 没  no | 力气  strength | Noun |
| 100 | 这辆坦克  This tank | 没  no | 炮弹  shells | Noun |

| 1 | 这家公司  This company | 没  not | 纳税  pay taxes | | Verb |
| --- | --- | --- | --- | --- | --- |
| 2 | 这个青年  This young man | 没  not | 睡觉  sleep | Verb | |
| 3 | 这个现象  This phenomenon | 没  not | 发生  happen | Verb | |
| 4 | 这种液体  This liquid | 没  not | 蒸发  evaporate | Verb | |
| 5 | 这个女生  This girl | 没  not | 毕业  graduate | Verb | |
| 6 | 这段时间  This period | 没  not | 浪费  waste | Verb | |
| 7 | 这篇文章  This article | 没  not | 抄写  transcribe | Verb | |
| 8 | 这份文件  This file | 没  not | 保存  save | Verb | |
| 9 | 这次会议  This conference | 没  not | 举行  hold | Verb | |
| 10 | 这批货物  These goods | 没  not | 过期  expire | Verb | |
| 11 | 这份试卷  This paper | 没  not | 批改  correct | Verb | |
| 12 | 这部作品  This work | 没  not | 印刷  print | Verb | |
| 13 | 这只兔子  This rabbit | 没  not | 跳  jump | Verb | |
| 14 | 这瓶可乐  This coke | 没  not | 打开  open | Verb | |
| 15 | 这位客人  This guest | 没  not | 离开  leave | Verb | |
| 16 | 这部电影  This film | 没  not | 播放  play | Verb | |
| 17 | 这封信件  This letter | 没  not | 收到  receive | Verb | |
| 18 | 这种情况  This situation | 没  not | 考虑  consider | Verb | |
| 19 | 这件衣服  This garment | 没  not | 洗  wash | Verb | |
| 20 | 这群候鸟  These migratory birds | 没  not | 回来  return | Verb | |
| 21 | 这项方案  This scheme | 没  not | 实行  implement | Verb | |
| 22 | 这个气球  This balloon | 没  not | 膨胀  swell | Verb | |
| 23 | 这面旗帜  This flag | 没  not | 悬挂  hoist | Verb | |
| 24 | 这只口哨  This whistle | 没  not | 吹响  blow | Verb | |
| 25 | 这个工人  This worker | 没  not | 做工  work | Verb | |
| 26 | 这只鹦鹉  This parrot | 没  not | 说话  speak | Verb | |
| 27 | 这次预算  This budget | 没  not | 增加  increase | Verb | |
| 28 | 这批物资  These materials | 没  not | 损耗  damage | Verb | |
| 29 | 这项任务  This task | 没  not | 处理  address | Verb | |
| 30 | 这条新闻  This news | 没  not | 轰动  splash | Verb | |
| 31 | 这列火车  This train | 没  not | 到达  arrive | Verb | |
| 32 | 这个苹果  This apple | 没  not | 消毒  sterilize | Verb | |
| 33 | 这个电话  This phone call | 没  not | 接听  answer | Verb | |
| 34 | 这颗炸弹  This bomb | 没  not | 掉落  drop | Verb | |
| 35 | 这本小说  This novel | 没  not | 阅读  read | Verb | |
| 36 | 这对男女  This couple | 没  not | 牵手  hold hands | Verb | |
| 37 | 这场疾病  This disease | 没  not | 恢复  recover | Verb | |
| 38 | 这次日食  This eclipse | 没  not | 观测  observe | Verb | |
| 39 | 这项规定  This provision | 没  not | 执行  enforce | Verb | |
| 40 | 这位犯人  This prisoner | 没  not | 逃脱  escape | Verb | |
| 41 | 这只乌鸦  This crow | 没  not | 喝水  drink | Verb | |
| 42 | 这份才华  This talent | 没  not | 埋没  bury | Verb | |
| 43 | 这台电脑  This computer | 没  not | 运行  run | Verb | |
| 44 | 这把椅子  This chair | 没  not | 组装  assemble | Verb | |
| 45 | 这枚电池  This battery | 没  not | 漏电  leak | Verb | |
| 46 | 这场大雨  This heavy rain | 没  not | 停止  stop | Verb | |
| 47 | 这场大火  This fire | 没  not | 蔓延  spread | Verb | |
| 48 | 这种水稻  This rice | 没  not | 杂交  hybridize | Verb | |
| 49 | 这个图形  This graphic | 没  not | 涂改  alter | Verb | |
| 50 | 这座大山  This mountain | 没  not | 倒塌  collapse | Verb | |
| 51 | 这块巨石  This boulder | 没  not | 移动  move | Verb | |
| 52 | 这颗子弹  This bullet | 没  not | 爆炸  explode | Verb | |
| 53 | 这位司机  This drive | 没  not | 违法  break laws | Verb | |
| 54 | 这位演员  This actor | 没  not | 微笑  smile | Verb | |
| 55 | 这块手表  This watch | 没  not | 转动  work | Verb | |
| 56 | 这个老头  This old man | 没  not | 咳嗽  cough | Verb | |
| 57 | 这堆木柴  These firewoods | 没  not | 燃烧  burn | Verb | |
| 58 | 这块木头  This wood | 没  not | 腐烂  rot | Verb | |
| 59 | 这群孩子  These children | 没  not | 做操  do gymnastics | Verb | |
| 60 | 这只山羊  This goat | 没  not | 吃惊  surprise | Verb | |
| 61 | 这个男人  This man | 没  not | 登山  climb mountains | Verb | |
| 62 | 这支股票  This stock | 没  not | 补仓  call margin | Verb | |
| 63 | 这份报告  This report | 没  not | 打印  print | Verb | |
| 64 | 这个计划  This plan | 没  not | 实施  implement | Verb | |
| 65 | 这些群众  These masses | 没  not | 抗议  protest | Verb | |
| 66 | 这个足球  This football | 没  not | 旋转  spin | Verb | |
| 67 | 这个老师  This teacher | 没  not | 讲课  teach | Verb | |
| 68 | 这种习惯  This habit | 没  not | 养成  form | Verb | |
| 69 | 这家银行  This bank | 没  not | 破产  rupt | Verb | |
| 70 | 这套工具  These tools | 没  not | 丢失  lost | Verb | |
| 71 | 这个球员  This player | 没  not | 犯规  foul | Verb | |
| 72 | 这种细菌  This bacterium | 没  not | 繁殖  multiply | Verb | |
| 73 | 这架飞机  This plane | 没  not | 起飞  take off | Verb | |
| 74 | 这头狮子  This lion | 没  not | 捕猎  hunt | Verb | |
| 75 | 这个孩子  This child | 没  not | 摔倒  fall | Verb | |
| 76 | 这块玻璃  This glass | 没  not | 破裂  break | Verb | |
| 77 | 这枚导弹  This missile | 没  not | 发射  launch | Verb | |
| 78 | 这个大妈  This auntie | 没  not | 跳舞  dance | Verb | |
| 79 | 这桶冰块  These ice cubes | 没  not | 融化  melt | Verb | |
| 80 | 这个手机  This phone | 没  not | 激活  activate | Verb | |
| 81 | 这座火山  This volcano | 没  not | 喷发  erupt | Verb | |
| 82 | 这块积木  This brick | 没  not | 安装  assemble | Verb | |
| 83 | 这批材料  These materials | 没  not | 采购  purchase | Verb | |
| 84 | 这个演员  This actor | 没  not | 签约  sign contracts | Verb | |
| 85 | 这个男生  This boy | 没  not | 抽烟  smoke | Verb | |
| 86 | 这只猴子  This monkey | 没  not | 点头  nod | Verb | |
| 87 | 这头鲸鱼  This whale | 没  not | 张嘴  open mouth | Verb | |
| 88 | 这把菜刀  This knife | 没  not | 生锈  rust | Verb | |
| 89 | 这幅油画  This painting | 没  not | 拍卖  auction | Verb | |
| 90 | 这面墙壁  This wall | 没  not | 粉刷  paint | Verb | |
| 91 | 这袋盐水  This saline | 没  not | 注射  inject | Verb | |
| 92 | 这个把手  This handle | 没  not | 晃动  shake | Verb | |
| 93 | 这块球场  This court | 没  not | 占用  occupy | Verb | |
| 94 | 这只蚯蚓  This earthworm | 没  not | 蠕动  squirm | Verb | |
| 95 | 这块钢板  This steel plate | 没  not | 收缩  shrink | Verb | |
| 96 | 这盏台灯  This lamp | 没  not | 发光  emit light | Verb | |
| 97 | 这只螃蟹  This crab | 没  not | 脱壳  shed shell | Verb | |
| 98 | 这台电视  This TV | 没  not | 报废  scrap | Verb | |
| 99 | 这个婴儿  This infant | 没  not | 哭闹  cry | Verb | |
| 100 | 这项协议  This agreement | 没  not | 废除  annul | Verb | |

**Table S2.** Static results for P200, N400 and P600 on written Chinese understanding.

Top: mixed-effects regression models. Bottom: simple effects test.

|  | **P200** | | | | **N400** | | | | **P600** | | | |
| --- | --- | --- | --- | --- | --- | --- | --- | --- | --- | --- | --- | --- |
|  | ***β*** | *Std.Error* | *t* | ***p*** | ***β*** | *Std.Error* | *t* | ***p*** | ***β*** | *Std.Error* | *t* | ***p*** |
| **POS** (verbs / nouns) | -0.519 | 0.363 | -1.43 | 0.158 | 0.534 | 0.182 | 2.939 | 0.005 | -0.476 | 0.211 | -2.254 | 0.028 |
| **L2Level** (low / mid / high) | 0.093 | 0.131 | 0.711 | 0.479 | 0.085 | 0.072 | 1.176 | 0.242 | 0.004 | 0.072 | 0.048 | 0.961 |
| **POS × L2Level** | 0.518 | 0.168 | 3.083 | 0.003 | -0.419 | 0.084 | -4.987 | <0.001 | 0.407 | 0.098 | 4.162 | <0.001 |

|  | **P200** | **N400** | **P600** |
| --- | --- | --- | --- |
|  | ***p*** | ***p*** | ***p*** |
| **L2Level within verb** | < .001 | < .001 | < .001 |
| **L2Level within noun** | .741 | .125 | .686 |
| **POS within low L2Level** | .566 | .273 | .468 |
| **POS within mid L2Level** | .01 | .027 | .022 |
| **POS within high L2Level** | .004 | < .001 | < .001 |

**Table S3.** Static results for P200, N400 and P600 on Chinese Sign Language understanding

(mixed-effects regression models).

|  | **P200** | | | | **N400** | | | | **P600** | | | |
| --- | --- | --- | --- | --- | --- | --- | --- | --- | --- | --- | --- | --- |
|  | ***β*** | *Std.Error* | *t* | ***p*** | ***β*** | *Std.Error* | *t* | ***p*** | ***β*** | *Std.Error* | *t* | ***p*** |
| **POS** (verbs / nouns) | 0.05 | 0.082 | 0.609 | 0.545 | -0.04 | 0.217 | -0.182 | 0.856 | 0.13 | 0.222 | 0.585 | 0.559 |
| **L2Level** (low / mid / high) | 0.023 | 0.028 | 0.818 | 0.415 | 0.101 | 0.071 | 1.424 | 0.157 | -0.097 | 0.073 | -1.336 | 0.184 |
| **POS × L2Level** | 0.014 | 0.038 | 0.377 | 0.707 | -0.044 | .01 | -0.434 | 0.665 | 0.032 | 0.103 | 0.309 | 0.758 |


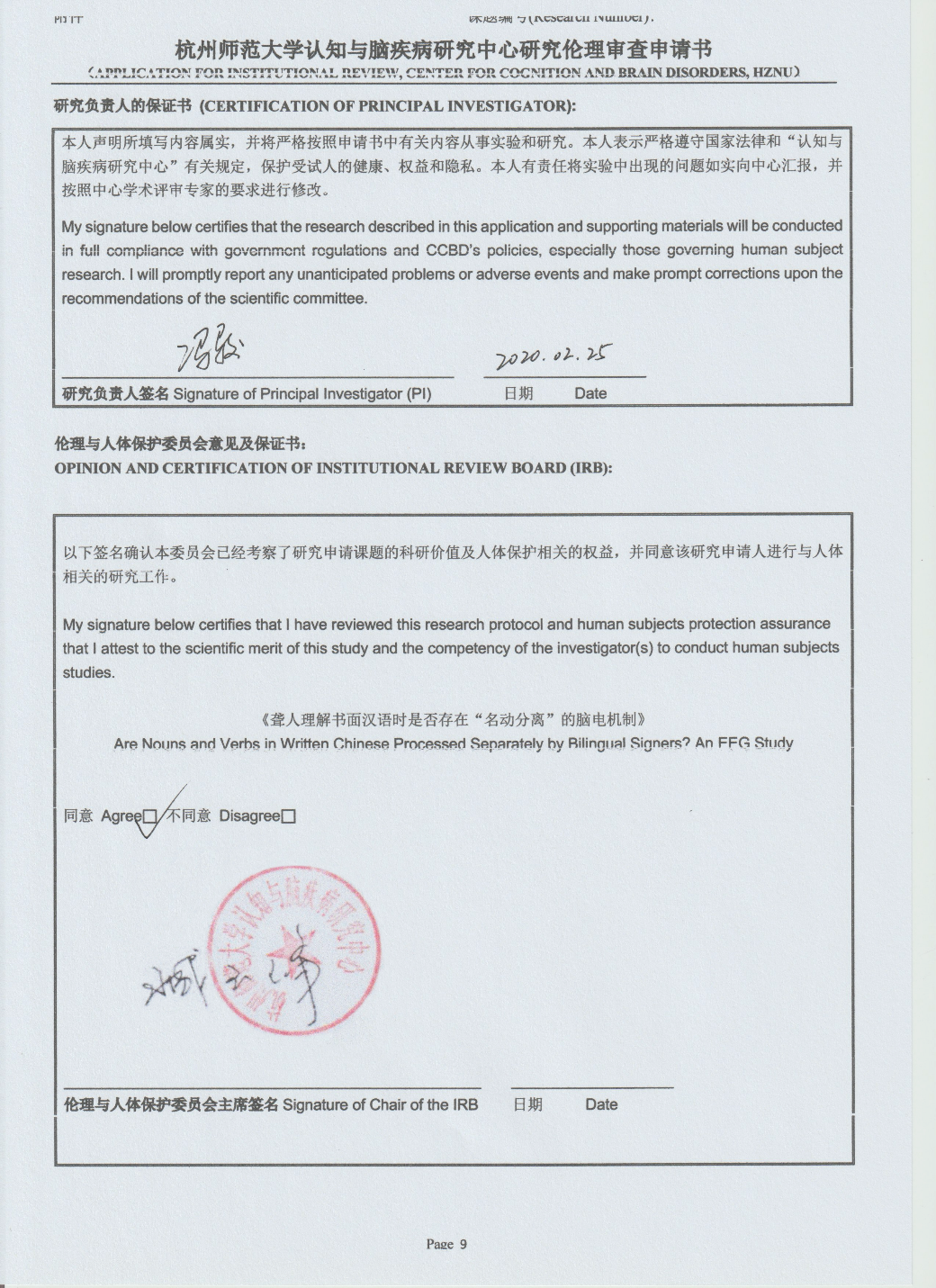

Supplement: Supplementary file 1 [file Table_1.docx]
